# Editorial: The interplay between microbiota and allergen in shaping respiratory mucosa immunity: Role in development of asthma

**DOI:** 10.3389/fimmu.2023.1131215

**Published:** 2023-02-16

**Authors:** Zhenwei Xia, Wenwei Zhong, Min Wu, Scott E. Evans

**Affiliations:** ^1^ Department of Pediatrics, Ruijin Hospital affiliated to Shanghai Jiao Tong University School of Medicine, Shanghai, China; ^2^ Department of Pediatrics, Xinhua Hospital affiliated to Shanghai Jiao Tong University School of Medicine, Shanghai, China; ^3^ School of Medicine and Health Sciences, Department of Biomedical Sciences, University of North Dakota, Grand Forks, ND, United States; ^4^ Department of Pulmonary Medicine, University of Texas MD Anderson Cancer Center, Houston, TX, United States

**Keywords:** asthma, airway inflammation, invariant natural killer cells, β2-adrenergic receptors, antibiotics, dendric cells

Asthma is a heterogeneous disease characterized by chronic airway inflammation and bronchial hyperresponsiveness. The pathogenesis of asthma is complex and far from fully understood. It is believed that interactions between genetic backgrounds and environmental factors lead to immune imbalance and chronic airway inflammation and play a pivotal role in the pathogenesis of asthma. Basic and clinical research studies reveal that dysbiosis of airway and/or gut microbiota can shape local immune responses that lead to asthma onset or alter asthma endotypes. In this issue, articles investigate the underlying mechanisms of asthma development using bulk RNA-sequencing (RNA-seq) or single-cell RNA-seq methods in clinical and animal model settings with emphasis on the relationship between microbes and asthma development.

Li et al. demonstrated that pre-treatment of mice with antibiotics to deplete the gut microbiota can alleviate OVA-induced allergic airway inflammation in an animal model of asthma. They identified invariant natural killer (iNKT) cells as an important target of investigation, as antibiotic pre-treatment reduced the numbers of iNKT cells in the lung tissue and draining lymph nodes. Several studies have previously demonstrated that iNKT cells participate in the pathology of allergic airway inflammation mainly by producing Th2 cytokines, such as IL-4, promptly and robustly upon activation. Li et al.’s study indicated that iNKT cell expansion and their function could be affected when gut microbiota composition is altered or absent, which may affect Th2 immune response and allergic airway inflammation (1). Another possible mechanism by which antibiotics alleviate allergic airway inflammation is *via* β2-adrenergic receptors (ADRB2)-expressing immune cells, especially ADRB2^+^ dendritic cells (DCs). Although ADRB2 is principally expressed on airway smooth muscle cell, a variety of immune cells such as DCs express ADRB2 (2). Activation of ADRB2 on airway smooth muscle cell causes bronchodilation. As such, ADRB2 agonists are widely used in asthma management. However, frequent use of short-acting ADRB2 agonists without concurrent use of inhaled corticosteroid (ICS) is associated with decreased responsiveness to ADRB2 agonist therapy, and may lead to increased risk of acute asthma exacerbations. Activation of ADRB2 on immune cells is associated with inflammation (2). In Li et al.’s study, *Adrb2* and *Adrb3* were demonstrated to be upregulated in the lungs of OVA-sensitized and challenged mice by flow cytometry and immunofluorescence when compared to those in the lungs of control mice. The ADRB antagonist propranolol alleviated Th2 immune response and allergic airway inflammation. Additional experiments indicated that ADRB2^+^DCs were involved in the allergic airway inflammation. This study indicated that, in addition to downregulation of ADRB2 expression on airway smooth muscle cells, persistent activation of ADRB2+ immune cells may be an important mechanism to explain the exacerbation risk of short-acting ADRB2 agonists without ICS. One concern raised by this study is that, although they demonstrated that antibiotic pre-treatment alleviates allergic airway inflammation and upregulation of *Adrb2* and *Adrb3* at the RNA level, they failed to detect Adrb2 and Adrb3 expression at the protein level, especially ADRB2^+^DCs number in lung tissues or draining lymph nodes. More studies are required to confirm these results.

Allergic rhinitis (AR) and asthma are both common airway allergic diseases with similar pathology profiles. It is well known that AR and asthma are closely related and frequently coexist with similar atopic background. However, the underlying mechanism of asthma development in AR patients remains unclear. In this issue, Li et al. compared the transcriptome features of patients with AR and asthma (AR-asthma) group and a group with AR alone using RNA-seq. They found that antimicrobial humoral immune response-related genes were upregulated in the AR-asthma group vs. the AR group during and outside of pollen season. *DEFA3*, *DEFA4*, *ELANE* and *LTF*, which encode antimicrobial peptides in neutrophils, were the most differently expressed genes (DEGs). Considering neutrophils are also regarded as important participants in the pathogenesis of asthma (3) and clinical studies revealed that AR patients with allergic asthma show higher levels of neutrophils, neutrophil chemotaxis and IL-8 in the sputum than patients without asthma, both at baseline and after bronchial allergen challenge (4), this study indicated that inflammation pathway mediated by neutrophils may be involved in asthma development in AR patients. However, considering the small sample of this study, more intensive studies are required to confirm these results.

In summary, the results from these studies provide some clues for us to further explore the pathogenesis of asthma, especially involvement of gut microbiota in development of asthma.

## Author contributions

ZX and WZ drafted the editorial, MW and SE reviewed and edited the editorial. All authors contributed to the article and approved the submitted version.
